# A single mutation in the core domain of the *lac* repressor reduces leakiness

**DOI:** 10.1186/1475-2859-12-67

**Published:** 2013-07-08

**Authors:** Pietro Gatti-Lafranconi, Willem P Dijkman, Sean RA Devenish, Florian Hollfelder

**Affiliations:** 1 Department of Biochemistry, University of Cambridge, Cambridge CB2 1GA, UK

**Keywords:** Lactose repressor, Protein engineering, Mutagenesis, Differential scanning fluorimetry, Protein production, Synthetic biology, Gene regulation, LacI

## Abstract

**Background:**

The *lac* operon provides cells with the ability to switch from glucose to lactose metabolism precisely when necessary. This metabolic switch is mediated by the *lac* repressor (LacI), which in the absence of lactose binds to the operator DNA sequence to inhibit transcription. Allosteric rearrangements triggered by binding of the lactose isomer allolactose to the core domain of the repressor impede DNA binding and lift repression. In Nature, the ability to detect and respond to environmental conditions comes at the cost of the encoded enzymes being constitutively expressed at low levels. The readily-switched regulation provided by LacI has resulted in its widespread use for protein overexpression, and its applications in molecular biology represent early examples of synthetic biology. However, the *leakiness* of LacI that is essential for the natural function of the *lac* operon leads to an increased energetic burden, and potentially toxicity, in heterologous protein production.

**Results:**

Analysis of the features that confer promiscuity to the inducer-binding site of LacI identified tryptophan 220 as a target for saturation mutagenesis. We found that phenylalanine (similarly to tryptophan) affords a functional repressor that is still responsive to IPTG. Characterisation of the W220F mutant, LacI^WF^, by measuring the time dependence of GFP production at different IPTG concentrations and at various incubation temperatures showed a 10-fold reduction in leakiness and no decrease in GFP production. Cells harbouring a cytotoxic protein under regulatory control of LacI^WF^ showed no decrease in viability in the early phases of cell growth. Changes in responsiveness to IPTG observed *in vivo* are supported by the thermal shift assay behaviour of purified LacI^WF^ with IPTG and operator DNA.

**Conclusions:**

In LacI, long-range communications are responsible for the transmission of the signal from the inducer binding site to the DNA binding domain and our results are consistent with the involvement of position 220 in modulating these. The mutation of this single tryptophan residue to phenylalanine generated an enhanced repressor with a 10-fold decrease in leakiness. By minimising the energetic burden and cytotoxicity caused by leakiness, LacI^WF^ constitutes a useful switch for protein overproduction and synthetic biology.

## Background

Cellular metabolism has evolved to be energy efficient, controllable and responsive to changes. The well-studied *lac* operon is an example of how cells respond to environmental conditions to maximise their use of energy resources [1-3]. In particular, the transcription repressor LacI plays a pivotal role: in the absence of the energy source lactose, LacI binds to the repressor binding site (‘operator DNA’) and inhibits the transcription of genes required for lactose metabolism [4,5]. In the absence of glucose (that acts as an inhibitor for the expression of genes encoded in the *lac* operon), binding of the lactose isomer allolactose triggers the release of LacI from the operator DNA *via* an allosteric mechanism [4,6]. This process shows the remarkable degree of coordination achieved during evolution: i) protein production is triggered by allolactose, which is itself formed from lactose by β-galactosidase; ii) LacI regulates the expression of proteins essential for uptake (by lactose permease) as well as metabolism (by a group of enzymes that includes β-galactosidase) of lactose; iii) the proteins encoded by the *lac* operon are constitutively expressed at low levels despite the high affinity of the interaction between LacI and its operator DNA (*K*_*d*_ = 10 pM for the O^1^ sequence [7]); iv) the basal expression of the *lac* operon, referred to as *leakiness*, is important for the function of the regulatory system: the low levels of permease and β-galactosidase that accumulate due to leakiness are needed to generate enough allolactose to cause switching of LacI [1].

LacI is a popular choice for protein overexpression: combined with the high-levels of transcription afforded by RNA polymerases from phages [8], LacI has been employed to uncouple the growth of the producing host from protein production (usually in *Escherichia coli*, but also in other organisms, including mammalian cells) [9,10]. The pET/BL21 system, originally established in 1990 [11-13], is still widely used for protein overproduction. The key features that are necessary for the biological role of LacI in the regulation of the *lac* operon are also the main limitations for its use in protein production. Attempts to remedy the metabolic burden and the cytotoxicity caused by leakiness in LacI-based expression systems include the introduction of additional control elements in the cell (increased LacI expression levels [14], co-expression of lysozyme [15], dual vectors [16], inducible phage RNA polymerase in BL21-AI cells [17]) or altered fermentation conditions such as variations of temperature [18] and media composition [19]. A comprehensive analysis of the challenges of protein overproduction in *E*. *coli* can be found elsewhere [20-24].

**Figure 1:**
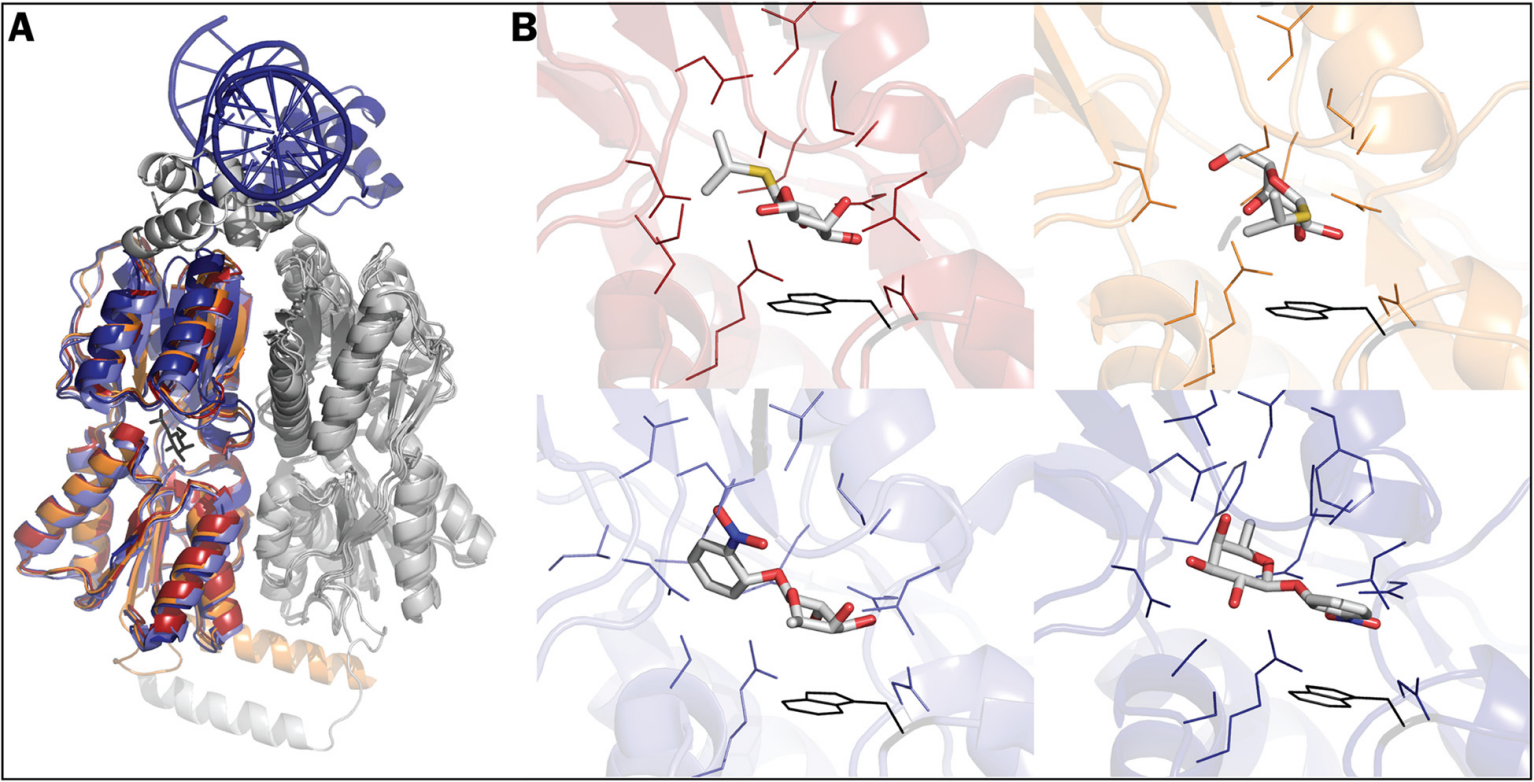
**Binding promiscuity in the inducer binding pocket of LacI. A**. Superposition of LacI PDB structures [PDB:2P9H] (dark red), [PDB:1LBH] (orange), [PDB:2PAF] (light blue) and [PDB:1EFA] (dark blue) showing the three-domain architecture of the protein. The second monomer of each structure is coloured in grey. IPTG (from structure 2P9H) is shown in black sticks. **B**. LacI binding pocket with the small molecule effectors IPTG (2P9H and 1LBH) and ONPF (2PAF and 1EFA). Interacting side chains as calculated by the DistancesRH pymol script (see Additional file 1: Table S1 for details) are shown. Structures follow the same colour-code and order as in panel **A**. The side chain of tryptophan 220 is depicted in black. Note how 1LBH and 1EFA show IPTG and ONPF, respectively, bound in opposite orientations to those seen in 2P9H and 2PAF.

The LacI protein consists of two domains that bind the operator DNA and the inducer (N-terminal and core domain, respectively), a short C-terminal domain required for the formation of the homo-tetramer and a hinge region between DNA and inducer binding domains (Figure 1A) [25,26]. The protein has two equilibrium states characterised by different degrees of secondary structure in the DNA binding domain and hinge region [27-29]. Binding of the inducer to the core domain stabilises the non-binding conformation, characterised by local unfolding in the DNA binding domain (up to 40 Å away) that decreases specificity for the *lac* operator and relieves the repression of transcription [30]. Crystallisation in the presence of both inducers and anti-inducers (IPTG and ONPF) has revealed the interaction networks in the binding pocket that correlate with either repression or de-repression [31], and mutational analysis has confirmed the importance of these amino acids [32,33]. However, the effects of random mutations [34-36] and insertions [37] in the LacI structure as well as targeted mutations in the hinge region [38] showed that the roles played by individual amino acids in long-distance allosteric transmission are still incompletely understood. Furthermore, the construction of chimeric repressors showed that specificity and function could be retained by the individual domains despite “mismatched interfaces” [39]. This observation points to intra-domain connectivity as the source of allosteric control. Protein engineering has brought about LacI variants with altered inducibility: directed evolution yielded variants with 100-fold tighter affinity for IPTG that carried mutations in both the DNA and inducer binding domains [40]. Similarly, mutations at positions 125 and 149 showed that changes at the inducer binding site influence the allosteric behaviour of LacI [32]. Mutants that better repress transcription were obtained by directed evolution, but required the co-evolution of a new operator DNA sequence [41].

The inducer binding site of LacI shows a remarkable degree of plasticity and binding promiscuity (see Figure 1B). Analysis of the amino acids in close proximity to the inducer/anti-inducer molecule in four crystal structures (Additional file 1: Table S1) indicates the existence of a large overlap between the residues involved in the binding of the two compounds. Among the closest amino acids, the tryptophan residue at position 220 establishes a large number of interactions with both substrates, in both orientations. Although this observation can partially be explained by the size of its bulky side chain, an aromatic group at this position has been reported to favour π-stacking in other repressors of the GalR/LacI family (PurR [42]) and might play a role in LacI as well (see ONPF binding in [PDB:1EFA], Figure 1B). We investigated this hypothesis by substituting the large and bulky tryptophan residue at position 220 with all 20 amino acids.

We built a reporter system in which the green fluorescent protein (GFP) is under the control of LacI in a standard pET vector. The effects of mutations and of fermentation conditions could then be monitored by measuring the amounts of GFP produced per cell *via* cytometry. Among tested variants, the LacI mutant W220F (LacI^WF^) shows a wild type phenotype with an increased dynamic range and a 10-fold reduction in leakiness. The characterisation of time-, IPTG concentration- and temperature-dependent GFP expression profiles together with a qualitative comparison of binding affinities by thermal shift analysis suggest that LacI^WF^ exerts more complete control of protein expression levels than LacI and limits the detrimental effect leakiness has on the host. LacI^WF^ will be a valuable tool for heterologous protein expression in *E*. *coli* due to its ability to reduce leakiness below detectable levels, its wider responsiveness to different IPTG concentrations and its compatibility with the traditional *lac* operator sequence.

## Results

### Saturation mutagenesis at position 220

**Figure 2:**
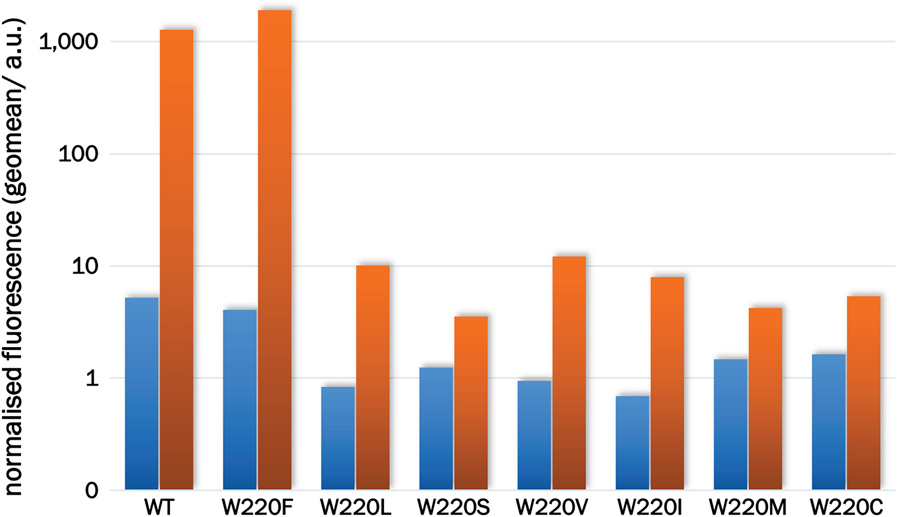
**LacI**^**WF **^**affords higher GFP expression levels and lower leakiness.** The expression of GFP was quantified by flow cytometry of *E*. *coli* cells carrying pLIGFP with the indicated mutations at position 220. Cells were incubated overnight at 37°C and the geometric mean of fluorescence measurements in the absence (blue bars) and presence (orange bars) of IPTG (0.01 mM) is displayed as arbitrary units (a.u.). To allow the comparison of data acquired on different days, fluorescence values have been normalised against WT (all mutants were tested under the same conditions and WT was measured each time). The original flow cytometry histograms are provided in Additional file 1: Figure S2.

Natural repressors of the LacI family [43,44] show variability in the type of the amino acid at position 220, with a non-exclusive preference for bulky, hydrophobic and aromatic side chains (Table 1). To test the effects on LacI function of mutations at position 220, saturation mutagenesis was performed at this position by means of whole plasmid amplification with degenerate oligonucleotides bearing an NNS codon. *E*. *coli* BL21(DE3) colonies carrying a mutated LacI were grouped by their phenotype into the following categories: I^-^ for variants that have lost the ability to bind DNA, I^s^ for mutants that still bind the operator but are incapable of induction, and I^+^ for variants that are fully functional, so bind the operator in the absence of inducer and release repression in the presence of inducer [35]. Approximately half of the library showed the I^-^ phenotype, while ~35% displayed an I^s^ phenotype and the remaining ~15% of the library was of the I^+^ phenotype. Sequencing identified six amino acids that gave the I^s^ phenotype, whereas wild type Trp and Phe (found in other repressors of the GalR/LacI family [43]) were the only amino acids that led to DNA release upon IPTG addition (Table 1). Using flow cytometry, the GFP expression of cells bearing the sequenced LacI variants was determined, and it was found that mutant W220F (LacI^WF^) has twice the dynamic range (defined as the ratio between the fluorescence of induced and un-induced cell cultures) of identically treated WT after overnight incubation at 37°C (Figure 2). As expected, the I^s^ variants gave expression levels in the range of noise.

**Table 1:** Amino acids at position 220 in naturally occurring repressors and those identified from screening the LacI W220X library

**Naturally occurring**				**Identified after saturation mutagenesis**
W	LacI			**W**
F	EbgR	CytR	PurR	**F**
S	MalI			*S*
L	TreR			*L*
P	GalR	GalS		*V*
Y	FruR	ExuR		*I*
T	AraR			*M*
				*C*

Amino acids found in other repressors of the GalR/LacI family are listed in the left column. Amino acids that allow LacI to retain the ability to bind to its operator DNA, and thus repress expression, are listed in the right column (in bold or italics to indicate whether they confer the I^+^ or I^s^ phenotype, respectively).

### LacI W220F is an effective regulator with low basal expression levels

**Figure 3:**
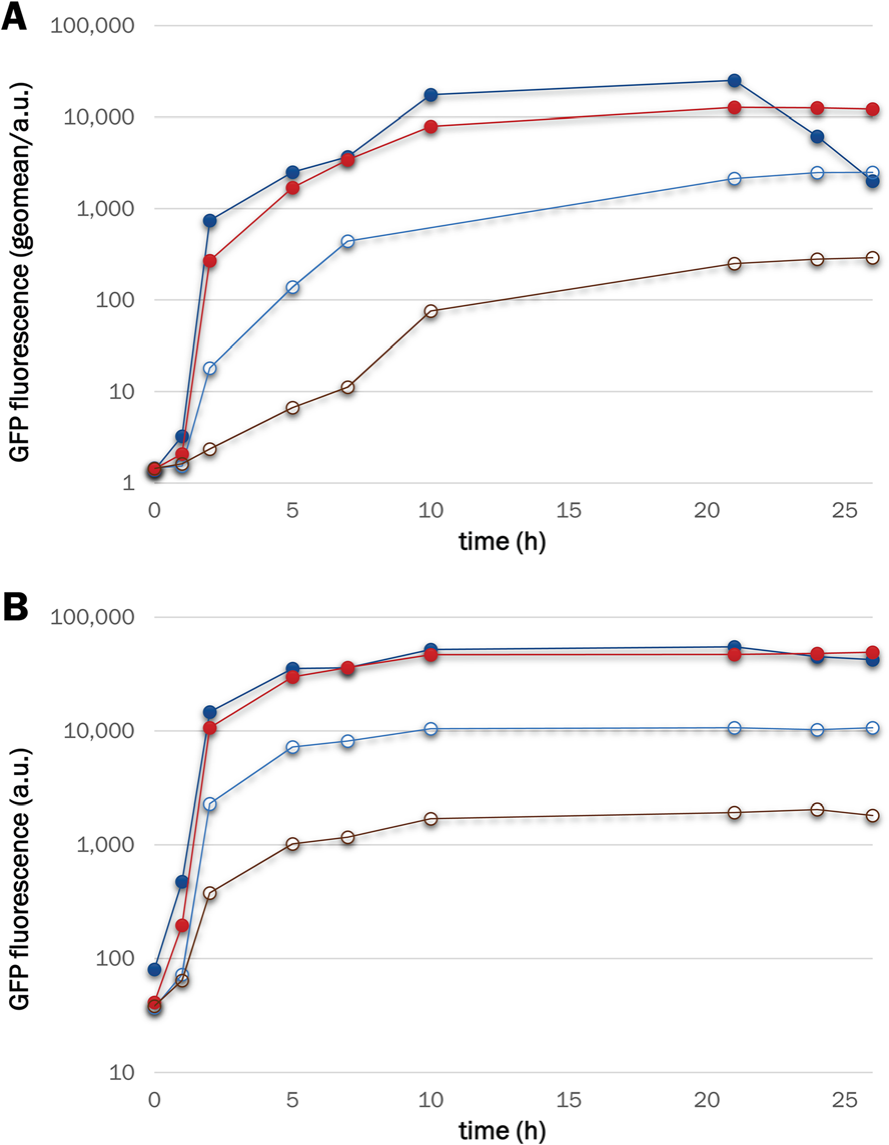
**GFP production kinetics for LacI and LacI**^**WF**^**.** Cells transformed with either pLIGFP (blue/cyan) or pLIGFP_W220F (red/brown) were grown at 37°C in the presence (solid symbols) or absence (open symbols) of IPTG. At regular intervals from the time of induction (time 0), the cell culture was sampled for FACS (panel **A**) or cell extract (panel **B**) analysis. The original flow cytometry histograms are provided in Additional file 1: Figure S3.

In LacI- and LacI^WF^-containing cells incubated at 37°C, similar GFP production rates were observed in the presence of IPTG, while 10-fold tighter repression was observed for cells bearing the mutant repressor in the absence of the inducer (compared to those bearing LacI, Figure 3A). Flow cytometry provides reliable population-level data on expression that is represented by the mean fluorescence of large datasets (> 30,000 events for each point in our case), although the cell to cell variation can be significant. Therefore, to confirm that the fluorescence measured by FACS is representative of the amounts of soluble and functional GFP that accumulates in the cell, cell suspension aliquots (1 mL) were pelleted, lysed and the fluorescence contained in the soluble protein fraction measured. As shown in Figure 3B, the same conclusions about the behaviour of the W220F mutation can be derived from the two types of analysis.

**Figure 4:**
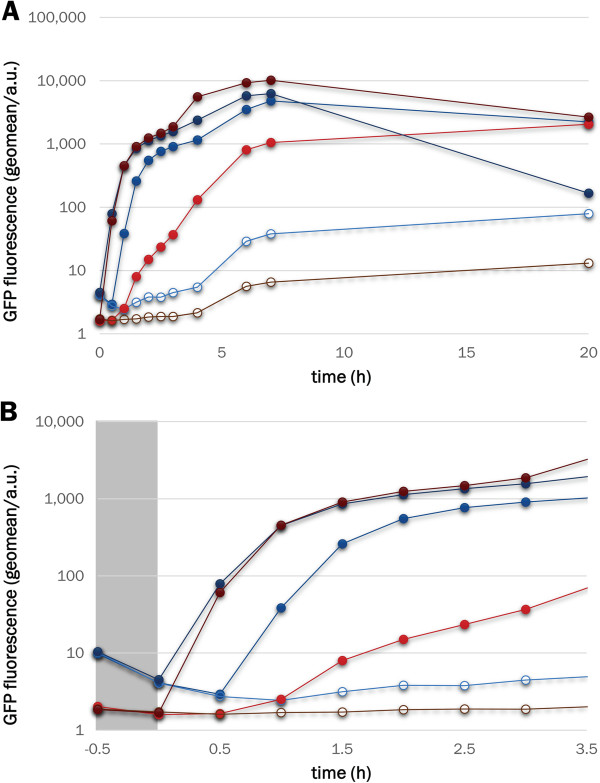
**LacI**^**WF **^**outperforms WT at 1 mM IPTG.** Cells bearing the WT repressor (dark blue/blue/cyan) or the W220F mutant (dark red/red/brown) were incubated in the absence (open symbols) of inducer or in the presence of either 1 mM or 0.01 mM IPTG (dark blue/dark red and blue/red, respectively). After overnight incubation (panel **A**), the GFP production levels of WT and mutant are indistinguishable by flow cytometry (but WT shows a marked decrease in fluorescence at the highest IPTG concentration). Although the final expression levels are similar, at early time points (panel **B**) and with 0.01 mM IPTG, LacI^WF^ shows a lag in induction relative to the WT. Background expression levels are consistently lower for the mutant, which shows no leakiness even before induction (note the different fluorescence values for blue and red lines in the grey-shaded area of panel **B**).

**Figure 5:**
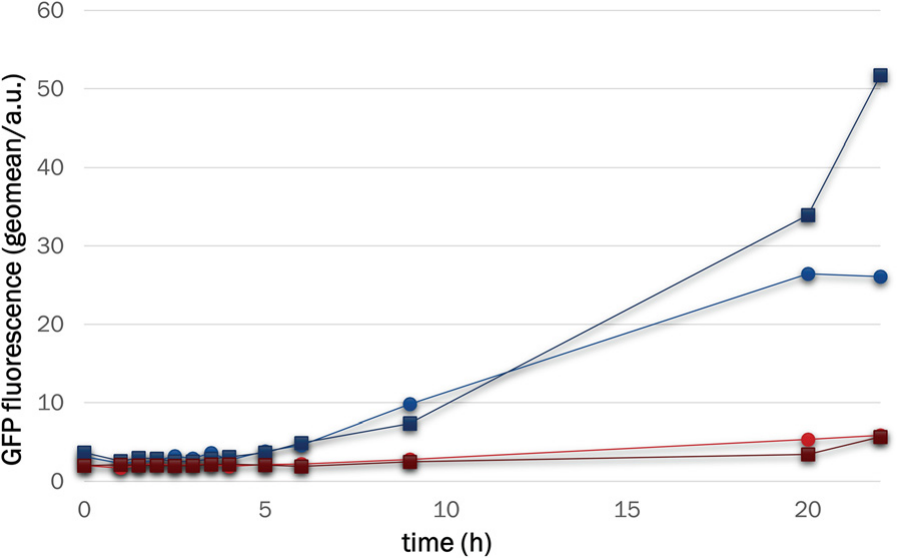
**Lowering incubation temperature from 37°C to 25°C does not affect leakiness in LacI**^**WF**^**.** GFP expression levels were measured on samples from cultures incubated without added IPTG at either 37°C (filled circles) or 25°C (filled squares) for both WT (dark blue/blue) and LacI^WF^ (dark red/red). For the mutant, expression levels in the absence of IPTG remain consistently 5-10 times lower than WT during the overnight incubation and are only marginally affected by temperature.

The primary objective of a protein over-expression system is to generate the highest possible amount of protein, although the ability to induce intermediate expression levels is sometimes desirable. While LacI-based systems produce large amounts of protein, control over the expression level is limited [24,45]. We thus tested the effects of different IPTG concentrations on the rates of GFP production (Figure 4A). If fluorescence values after an overnight incubation are compared, LacI^WF^ performs as well as WT at 0.01 mM IPTG and outperforms WT at 1 mM IPTG (as the mutant seems to benefit from a slower decay of expressed protein after the expression peak). Protein production rates in the early, linear phase (Figure 4B) are similar for LacI and LacI^WF^ at 1 mM IPTG, whereas at 0.01 mM inducer LacI^WF^ has a longer delay and slower rate of protein production. The observation of negligible GFP expression before induction (Figure 4B) further suggests that mutant W220F provides much tighter DNA binding in the absence of IPTG. When protein production is performed at the sub-optimal growth temperature of 25°C, the leakiness of WT is significantly reduced, but remains considerably above that of LacI^WF^ (Figure 5).

### Mutant W220F has an expanded dynamic range

**Figure 6:**
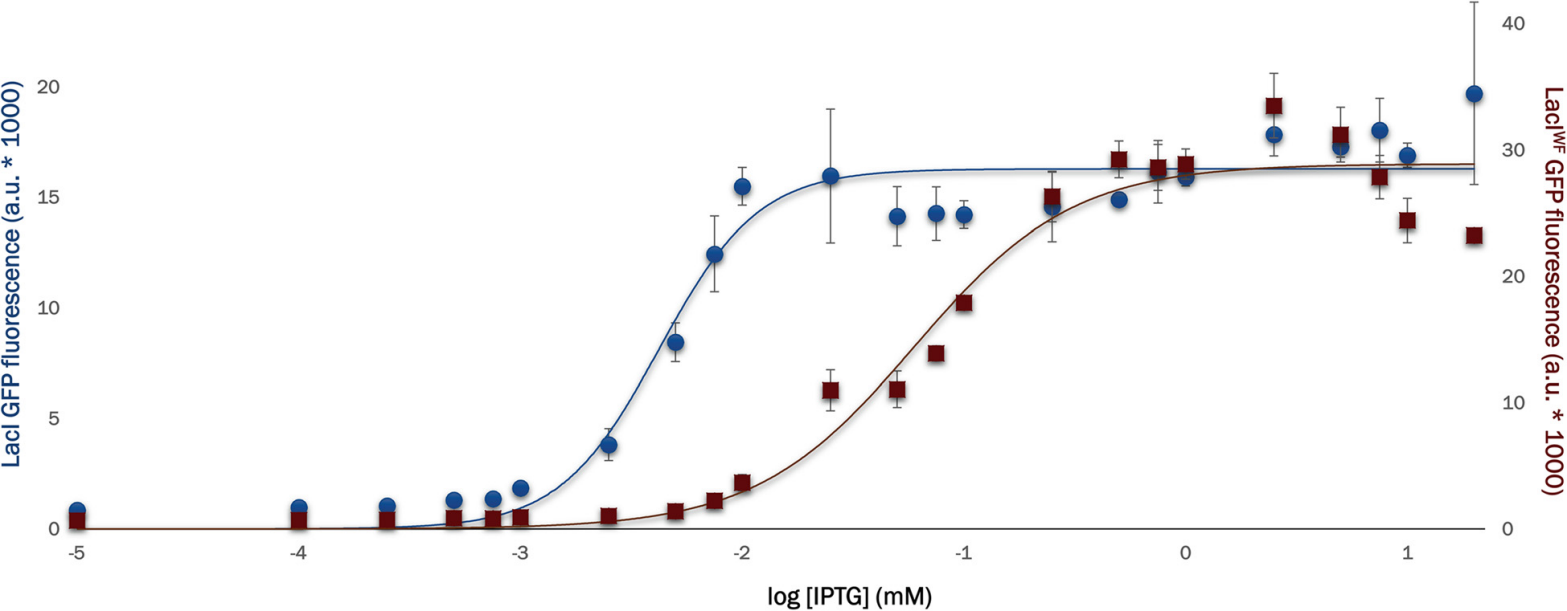
**LacI**^**WF **^**shows a wider dynamic range than WT.** GFP expression levels measured on 200 μL of cell culture bearing LacI (blue, left axis) or LacI^WF^ (red, right axis) after 24 h incubation at 37°C with different IPTG concentrations. For each variant, three independent repeats were corrected for cell density. The plot shows averaged values and standard deviation for each point. Curves have been drawn merely to guide the eye. The two datasets are plotted on different axes to show the difference in dynamic range between LacI and LacI^WF^.

The dependence of GFP expression on IPTG concentration was tested using an end-point assay [45]: cells were incubated in a multiwell-plate for 24 h with a range of IPTG concentrations after which the fluorescence of the cell suspension was measured directly. The rightward shift of the curve for LacI^WF^ in Figure 6 suggests that this mutant has lower affinity for IPTG than the WT protein. The shallower slope of the LacI^WF^ induction curve provides greater ‘tunability’ than offered by the WT protein, which switches completely from uninduced to fully induced within a 10-fold increase in IPTG concentration. In contrast, the W220F mutant spreads an equivalent increase in expression over a much wider IPTG concentration range, requiring more than a 100-fold increase in IPTG concentration to be fully induced. However, the shape of the titration curve depends heavily on growth conditions (see, for example, the effect of temperature in Additional file 1: Figure S1). The observation that a reduction in leakiness is accompanied by a wider dynamic range in LacI^WF^ (note the different y-axis scales in Figure 6) stands in contrast to mutants examined in previous reports [45]. At concentrations above ~ 0.1 mM IPTG, LacI^WF^ yields more protein than WT LacI.

**Figure 7:**
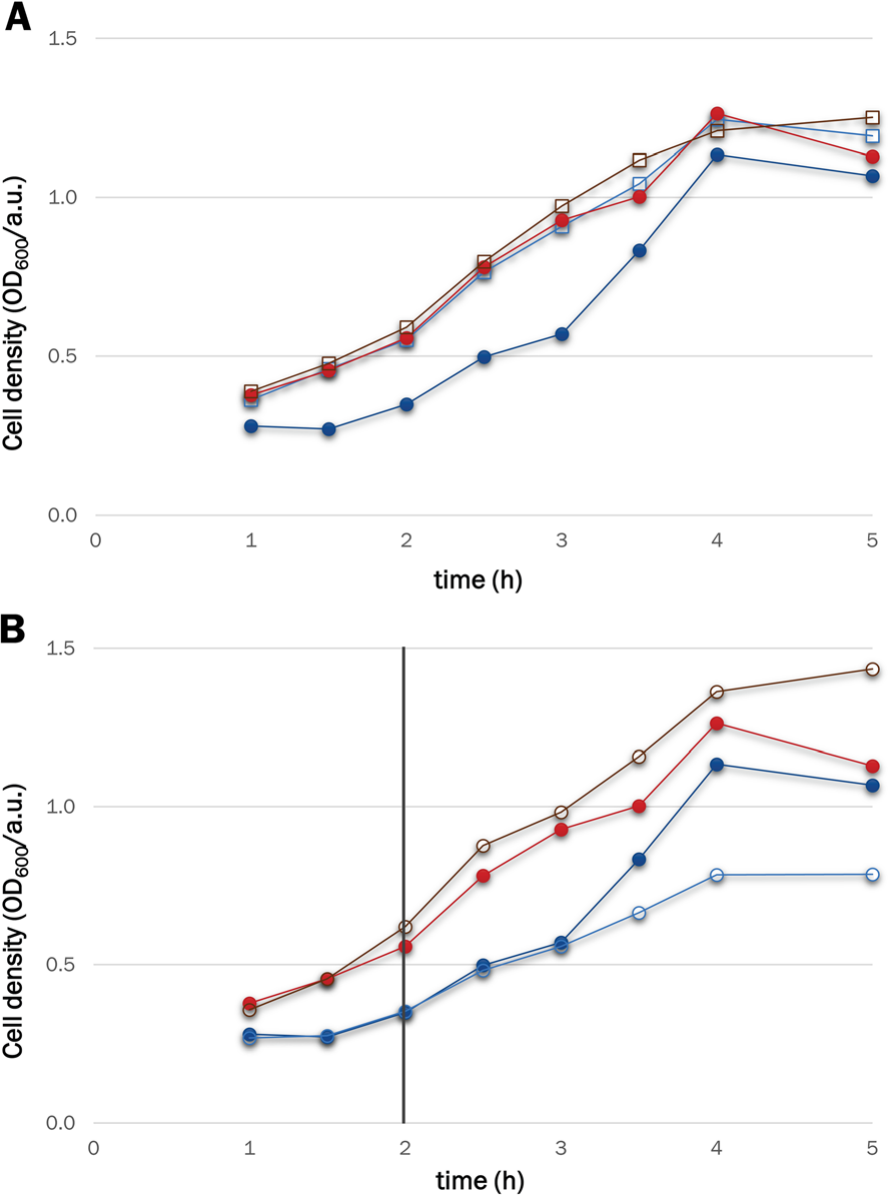
**LacI**^**WF **^**reduces the cytotoxicity of a plasmid encoding a MBP-HIV-protease. A**. Optical density of BL21(DE3) cell cultures bearing pLIGFP (blue line, open squares), pLIGFP_W220F (red line, open squares), pLIHP (blue line, solid circles) or pLIHP_W220F (red line, solid circles) in the absence of IPTG. **B**. Comparison of un-induced (filled circles) and IPTG-induced (open circles) cells expressing the MBP-HIV protease fusion under the control of LacI (light/dark blue) or LacI^WF^ (dark/light red). Cell cultures were induced after 2 hours (black vertical line).

To probe the effects of leaky expression at levels below those detectable by cytometry, we tested the growth rates of cells bearing HIV protease under the control of either WT LacI or LacI^WF^. A tethered dimer of HIV protease [46], which cannot be produced in soluble form under WT LacI control due to its toxicity [47,48], was fused to maltose-binding protein (MBP) and cloned in place of GFP to give plasmids pLIHP and pLIHP_W220F. In the absence of the inducer, cells transformed with the pLIHP_W220F plasmid showed growth curves that are indistinguishable from those of cells expressing GFP (Figure 7, panel A). In contrast, when the expression of the MBP-HIV protease is under the control of WT LacI, cells show slower growth rates. This observation indicates that the leakiness of LacI^WF^ is reduced to levels at which even the expression of the highly toxic HIV protease does not affect cell viability. When 0.01 mM IPTG is added to the cell culture, the growth of WT LacI is reduced further, while the growth of LacI^WF^ is not affected (Figure 7, panel B).

### Stability of purified LacI^WF^ corroborates *in vivo* results

**Figure 8:**
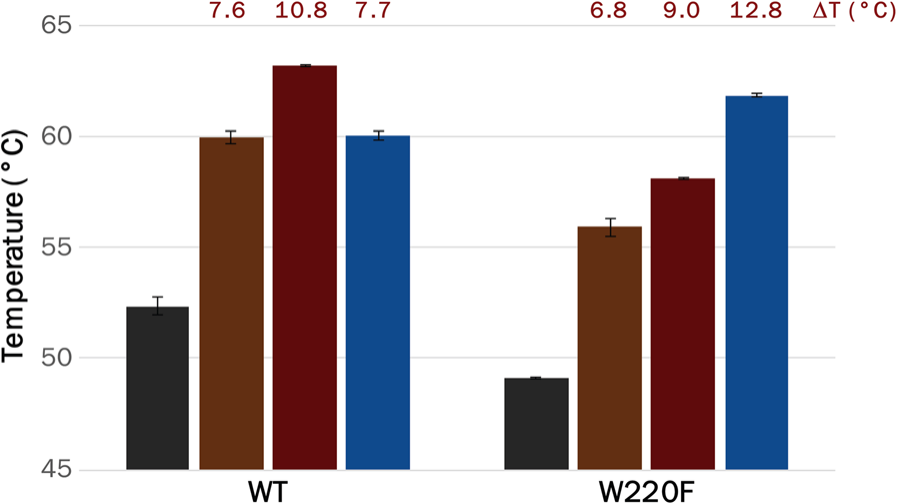
**LacI**^**WF **^**has increased stability in the presence of operator DNA.** Purified LacI and LacI^WF^ were incubated at a final concentration of 1 μM in the absence (grey) or presence of different concentrations of IPTG (brown and dark red for 1 mM and 10 mM, respectively) or operator DNA (2.5 μM, blue) and their unfolding temperatures measured by DSF. The increase in melting temperature caused by the presence of each ligand (ΔT) is reported above the corresponding bar.

The interaction between purified LacI (WT and LacI^WF^) and either inducer or operator DNA was probed by thermal shift assay. Thermal unfolding of purified proteins was measured by differential scanning fluorimetry (DSF) in the presence of IPTG or of the operator. To this end, we expressed WT and LacI^WF^ as C-terminal His_6_-tag fusion proteins under the control of L-arabinose and affinity-purified both proteins. Purified LacI and LacI^WF^ showed melting temperatures of 52.4±0.3°C and 49.1±0.1°C, respectively, indicating a slight destabilisation of the mutant (Figure 8). The addition of 1 mM IPTG increases the melting temperature of WT by 7.6°C, whereas LacI^WF^ is stabilised to a slightly smaller extent (+6.8°C). A similar trend is observed with 10 mM IPTG (melting temperatures are 63.2±0.1°C and 58.1±0.1°C for WT and mutant, respectively), indicating that IPTG is still tightly bound by both enzymes, despite the lower stability of LacI^WF^. On the other hand, the addition of 2.5 μM operator DNA has a much larger effect on LacI^WF^, which is stabilised by 12.8°C (in contrast to the more modest 7.7°C increase in melting temperature for WT LacI). The smaller extent of stabilisation by IPTG in conjunction with better stabilisation by operator DNA is consistent with the reduction in leakiness and in IPTG sensitivity observed *in vivo*.

## Discussion

Advances in structural and synthetic biology require a better understanding of the consequences of protein (over)expression and the development of more adaptable production protocols. Despite the increase in efficiency and robustness of *in vitro* expression systems [49-51], bacteria (and *E*. *coli* in particular) still represent a very common choice for protein production [52]. The improvements in yields and efficiency delivered *via* metabolic engineering and synthetic biology [53-55] are complemented by more analytical approaches that aim to understand the properties that cause aggregation and its effects on host viability [56-59]. As a result of these efforts, a number of strains, fermentation conditions and co-expression partners (such as chaperones or rare tRNAs) can be used for high-throughput protein expression screening [60]. In particular, the ability to control protein expression is of relevance, as the ideal system would allow the user to choose whether yield, quality or reduced toxicity are to be prioritised.

Although several approaches have been explored to limit the basal expression rates of proteins under the control of LacI [21], reducing leakiness through changes in the functional properties of the repressor itself has proven to be remarkably difficult. Large-scale studies on mutant libraries have helped to understand the structural features that allow LacI to be efficient at repressing transcription and specific for both inducer and operator DNA [35-37], but have rarely led to the isolation of better mutants [40]. In LacI, long range communications are responsible for the transmission of the signal from the inducer binding side to the DNA binding domain [30], as suggested by the effects of mutations in the hinge region [38] and by the introduction of disulphide bonds at positions 125, 149 and 193 [32,33]. Our results show that saturation mutagenesis at position 220 generates the non-inducible I^s^ phenotype in half of the cases, whereas the ability to respond to IPTG is exclusive to aromatic side chains (tryptophan and phenylalanine, see Figure 2). Considering that binding of allolactose to LacI would not benefit from aromatic interactions, the presence of tryptophan at position 220 is likely the result of a compromise between tight binding of ligand and efficient repression. This view is consistent with the involvement of position 220 in modulating long-range communication between the inducer and DNA binding sites.

The *lac* operon provides cells with the ability to switch rapidly from glucose to lactose metabolism precisely when necessary. This ability to detect and respond to environmental conditions comes at the cost of the encoded enzymes being constitutively expressed at low levels, but the *lac* operon has found a way to balance energetic cost and fast response. While the *lac* operon in its natural context represents an impressive achievement of natural evolution, the ability of the *lac* repressor to uncouple transcription from cell growth has made it a useful resource in protein production, and its applications in molecular biology represent an early example of synthetic biology. Natural evolution has provided us with a useful tool; however, man-made systems require different properties that can be delivered by protein engineering and directed evolution [61-63]. The engineered repressor LacI^WF^ minimises the energetic burden prior to induction and growth inhibition by cytotoxic proteins and surpasses LacI in terms of maximal GFP expression. LacI^WF^ thus constitutes a useful switch for protein overproduction and synthetic biology.

## Conclusions

A single mutation, W220F, in the inducer-binding site of LacI generates a fully functional repressor (named LacI^WF^) with a 100-fold wider dynamic range at 37°C and a 10-fold decrease in leakiness under all conditions tested. The reduction in leakiness results in unimpaired growth when toxic proteins are cloned under the control of the mutant repressor. For IPTG concentrations above 0.1 mM, LacI^WF^ yields more GFP than WT LacI, possibly due to a decrease in the rate of protein production (in particular at low inducer concentrations). Data from thermal shift assays indicate changes in the stabilisation effects of IPTG and operator DNA on LacI^WF^ compared to WT LacI and, together with our *in vivo* analysis, are suggestive of altered affinities in the mutant. In conclusion, LacI^WF^ is compatible with the conventional operator sequence, and has both reduced leakiness and an improved dynamic range, so could be a superior alternative to the wild type repressor for controlling protein expression.

## Methods

### Chemicals and growth media

All chemicals were of analytical grade and were purchased from Sigma-Aldrich (unless otherwise stated). *E*. *coli* strain Top10 was used for all standard cloning procedures. Protein expression was carried out in the BL21(DE3) strain in Luria broth (LB) supplemented with 100 μg/mL ampicillin (LB-amp), agar (18 g/l) and IPTG, when required. For a full list of primers used, see Additional file 1: Table S2.

### Construction of plasmids

Plasmid pLIGFP was constructed by cloning the sequence for a green fluorescent protein (GFP) variant (bearing mutations S65G, V68L, S72A, Y203T and H231L, [64,65]) into a modified pET22b vector. First, the sequence of LacI was amplified with primers pLI01 and pLI02 to introduce the *Hind*III and *Nhe*I restrictions sites, respectively, at positions 181 and 988 (via silent mutations). This PCR product was purified and used as a megaprimer in a second PCR reaction to yield plasmid pLI. Subsequently, the GFP gene was amplified with primers pLI05 and pLI06 to introduce *Nde*I and *Xho*I sites, respectively and cloned into the double-digested pLI plasmid to give pLIGFP (plasmid pLIGFP_W220F, encoding for the LacI^WF^ mutant, is available from Addgene). Plasmids pLIHP and pLIHP_W220F were built following the same procedure: MBP-HIV protease fusion was amplified by PCR with primers pGMH2f and pGMH3r and cloned in place of GFP in plasmids pLIGFP and pLIGFP_W220F. All constructs were verified by sequencing. The standard PCR mix included 50 ng of template DNA, 250 nM of each dNTP, 0.5 μM of each primer, and 0.5 U of *PfuTurbo* polymerase (Agilent) in 20 μL of 1× *Pfu* buffer. Thermo-cycling consisted of 5 min at 95°C followed by 25 cycles of 30 sec at 95°C, 30 sec at 52°C and 1 min at 72°C, and a final extension step of 10 min at 72°C. For the megapriming reaction, the protocol was modified as follows: the PCR mix contained 40 ng of pET22b plasmid and 300 ng of the amplified LacI gene fragment in 50 μL of 1× *Pfu* buffer and the cycling procedure was altered so that annealing was carried out at 54°C for 45 sec and extension at 68°C for 8 min.

### W220X library construction

Full randomisation of position 220 was achieved by means of back-to-back primer PCR [66]. Full-plasmid amplification of pLIGFP was carried out with primers pLI07 (which contains the degenerate NNS codon) and pLI08 and the linear DNA amplicon was treated with T4 Polynucleotide Kinase (200 ng of DNA with 5 U of enzyme in 20 μL of 1× T4 Ligase buffer) for 30 min at 37°C. After lowering the temperature to 22°C, 400 U of T4 Ligase were added to the mix and the reaction was incubated for two additional hours. Aliquots of this reaction were then used directly for transformation of competent *E*. *coli* cells. The PCR reaction for full-plasmid amplification consisted of 60 ng pLIGFP, 250 nM of each dNTP, 0.5 μM of each primer, 1 U of *PfuTurbo* polymerase (Agilent) in 50 μL of 1× Cloned *Pfu* buffer. Thermo-cycling consisted of 10 min at 95°C followed by 25 cycles of 30 sec at 95°C, 30 sec at 60°C and 8 min at 72°C, and a final extension step of 10 min at 72°C.

### Growth conditions and fluorescence analysis

For all growth experiments, cells were pre-incubated overnight at 37°C, diluted 1:50 into 5–50 mL of fresh LB-amp, and then incubated at either 37°C or 25°C for up to 26 h. For cytometry-based fluorescence analysis, aliquots of 25 μL were sampled at the indicated times and stored in 1 mL of PBS containing 0.5% formaldehyde. For each experiment, at least 30,000 events for each sample were measured using a Becton Dickinson FACScan Flow Cytometer and analysed with Flowing Software. For consistency, data from a single representative experiment are plotted in each figure. Although this treatment does not allow derivation of error bars, values represent the average of > 30,000 events and errors are expected to be minimised. Responsiveness to different IPTG concentrations was measured by growing 1 mL cultures of BL21(DE3) cells in multiwell plates for 24 h at 37°C and then measuring OD_600nm_ and fluorescence (excitation 485 nm, emission 527 nm) with a M5 Spectramax microplate reader (Molecular Devices). To measure the fluorescence of cell extracts, aliquots (1 mL) were sampled at the indicated times, cells pelleted and frozen at −20°C prior to lysis in 200 mL of PBS containing BugBuster (1×) and Lysonase (Millipore). After the removal of cell debris by centrifugation, the fluorescence of the supernatant was measured in a plate reader.

### Arabinose-induced expression of LacI

Plasmid pBAD-LacI was constructed by cloning a modified LacI gene (amplified with primers pAL01 and pAL02) into a *Nco*I and *Sal*I double-digested pBADmyc-hisB plasmid and verified by sequencing. The resulting construct encodes for the *lac* repressor with a C-terminal His_6_-tag (plasmid pBAD-LacI is available from Addgene). Protein over-expression was carried out in Top10 cells by addition of 0.2% (w/v) L-arabinose. After 4 h of expression at 20°C, cells were harvested, resuspended in lysis buffer (25 mM Tris–HCl, 300 mM NaCl, 5 mM imidazole, pH 8) and 8 μL lysonase (Novagen) and lysed by sonication. After centrifugation, the soluble extract was affinity-purified on a 5 mL HisTrap FF purification column (GE Healthcare) following the manufacturer’s instruction and then buffer-exchanged into LacI storage buffer (200 mM Tris–HCl, 200 mM KCl, 10 mM EDTA, 3 mM DTT, pH 7.4) using two HiTrap buffer exchange columns (5 mL, GE Healthcare) in sequence.

### Differential scanning fluorimetry

Purified proteins were diluted in LacI storage buffer at the final concentration of 1 μM in the presence of 2× SYPRO Orange (Invitrogen) and, when indicated, either IPTG or the operator DNA (primers LacO1f and LacO1r, corresponding to the O^1^ sequence [7], pre-annealed) at the concentrations stated in the text. Melting curves [67] were recorded in triplicate over the range 25°C to 95°C (1°C/min increases) with a real-time PCR machine (Corbett Research Rotor-Gene 6000). Plotted values are the average of three replicates.

## Abbreviations

IPTG: Isopropyl-β-D-thiogalactopyranoside; ONPF: O-nitrophenyl-β-D-fucoside.

## Competing interests

The authors declare that they have no competing interests.

## Authors’ contributions

PG-L designed the project, carried out experiments, analysed data and wrote the paper. WPD performed preliminary experiments. SRAD contributed to project design and wrote the paper. FH directed the research and wrote the paper. All authors read and approved the final manuscript.

## Supplementary Material

Additional file 1: Table S1 Connectivity of residues in the binding pocket of LacI that interact with IPTG or ONPF. **Table S2.** List of primers. **Figure S1.** Dynamic range of LacI and LacI^WF^ at 25°C. **Figure S2.** Original cytometry data for Figure 2. **Figure S3.** Original cytometry data for Figure 3. Click here for file
