# Supplementary material for: A single mutation in the core domain of the lac repressor reduces leakiness
Source: Microb Cell Fact. 2013 Jul 8;12:67. doi: 10.1186/1475-2859-12-67 (PMC3722110; doi:10.1186/1475-2859-12-67)
Supplement: Additional file 1: Table S1 — Connectivity of residues in the binding pocket of LacI that interact with IPTG or ONPF. Table S2. List of primers. Figure S1. Dynamic range of LacI and LacIWF at 25°C. Figure S2. Original cytometry data for Figure 2. Figure S3. Original cytometry data for Figure 3. [file 1475-2859-12-67-S1.pdf]

A single mutation in the core domain of the *lac* repressor reduces leakiness.

Pietro Gatti-Lafranconi[1], Willem P Dijkman[1], Sean RA Devenish[1], Florian Hollfelder[1§]

[1]Department of Biochemistry, University of Cambridge, Cambridge CB2 1GA (UK)

[§]Corresponding author

Additional files

Table S1 – Connectivity of residues in the binding pocket of LacI that interact with IPTG or ONPF. 2

Table S2 – List of primers 3

Figure S1 – Response of WT LacI and LacI^{WF} to IPTG at 25 °C. 4

Figure S2 - Original cytometry data for Figure 2. 5

Figure S3 - Original cytometry data for Figure 3. 6

References 7

Table S1 – Connectivity of residues in the binding pocket of LacI that interact with IPTG or ONPF.

Distances between IPTG or ONPF and the binding site residues of LacI listed in column 1 were measured using the pymol script DistancesRH [1]. The number of atoms within 4.25 Å of any atom of the small molecules (IPTG or ONPF) are listed in columns 2-5. Higher scores in this classification suggest higher connectivity.

PDB ID	2P9H	1LBH	2PAF	1EFA
binder	IPTG	IPTG	ONPF	ONPF
SER/69	3	2		
LEU/73	4		3	2
ALA/75	5	6	2	6
PRO/76	3	2	1	2
ILE/79	1	2	3	2
ASN/125	3		10	2
PRO/127	1			
LEU/148			5	
ASP/149	7	1	10	11
ILE/160			1	
PHE/161			3	2
SER/191		1	5	1
SER/193	2			7
ARG/197	9	11	12	8
TRP/220	21	10	20	16
ASN/246	5	3	8	6
ASP/274	9	11	11	9
GLN/291	1	3	8	5
PHE/293				3
LEU/296				2

Table S2 – List of primers

primer name	sequence (5' – 3')
pLI01	CAAACAAAGCTTGCTGATTGGCGTTGC
pLI02	GGTTTGCGTATTGGGTGCTAGCGTGG
pLI05	GAAGGAGATATACATATGGGCAAAGTGAGCAAGGG
pLI06	GTGGTGGTGCTCGAGTTACTTGTAAAGCTCGTCCATGCC
pGMH2f	TCGTATATTAATGAAAATCGAAGAAGGTAAACTGG
pGMH3r	CTTTGTTAGCAGCCGGATCAAGC
pLI07	AAGGCGACNNSAGTGCCATGTCCG
pLI08	CCCGTTCCGCTATCGGCTG
pAL01	CTGAATCCCATGGTGAAACCAGTAACGCTATACGATGTCG
pAL02	TCAGTAGTCGACCTGCCCGCTTTCCAGTCGG
LacO1f	TGTTGTGTGGAATTGTGAGCGGATAACAATTTCACACAG
LacO1r	CTGTGTGAAATTGTTATCCGCTCACAATTCCACACAACA

Figure S1 – Response of WT LacI and LacI^{WF} to IPTG at 25 °C.

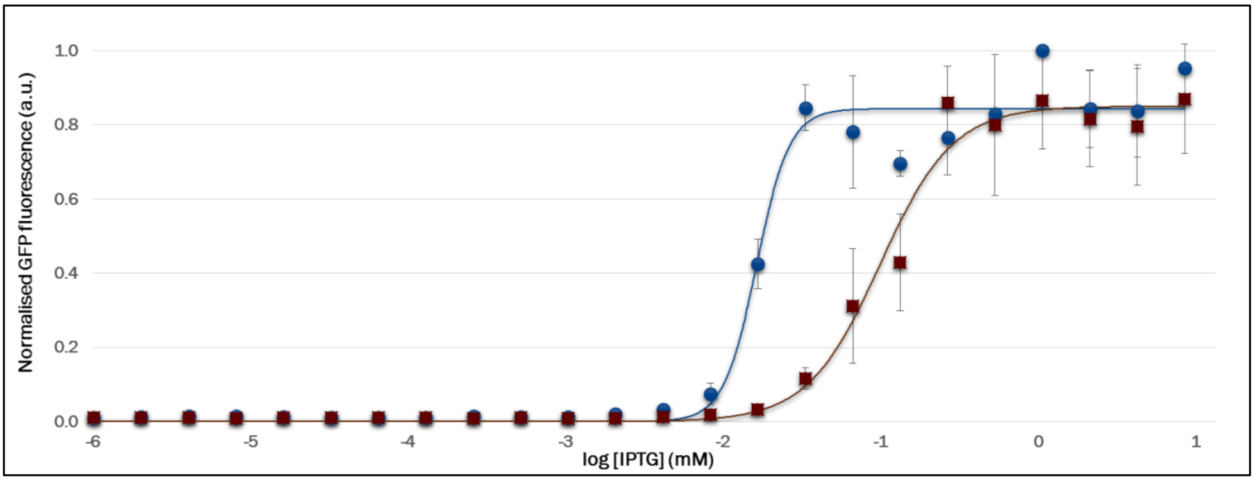

GFP expression levels measured on 200 μl of cell culture bearing LacI (blue) or LacI^{WF} (red) after 24 h incubation at 37 °C with different IPTG concentrations. The experiment was performed in an analogous way to Figure 6 and followed the previously published procedure [2]. For each variant, three independent repeats were corrected for cell density, normalised on the corresponding highest value and plotted as averages with standard deviation. The shift of the curve towards higher IPTG concentrations in the case of LacI^{WF} indicates the responsiveness of the mutant to IPTG is reduced even at 25 °C. FACS analysis (not shown) reveals that incubation at 25 °C lessens phenotypic variability within the cell culture and limits the toxicity of protein production at high IPTG concentration. Nevertheless, fluctuations between different experiments can still influence the slope of the curve, hence our choice to avoid the determination and discussion of EC50 values.

Figure S2 - Original cytometry data for Figure 2.

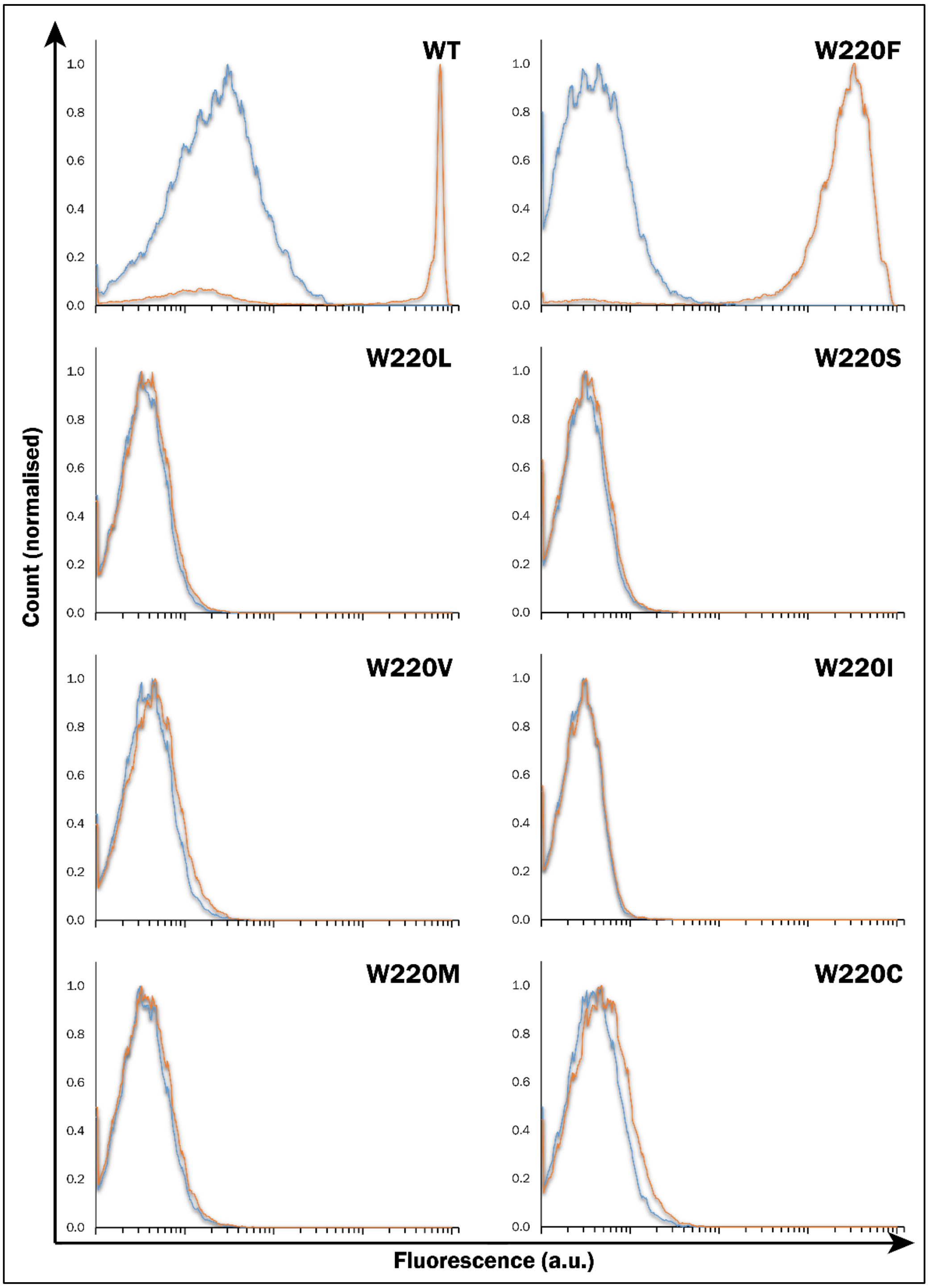

Raw cytometry data (used to build the chart in Figure 2) of the effects of different amino acids at position 220 on the responsiveness of the repressor to IPTG. Cells were measured after overnight incubation at 37 °C either with or without 0.01 mM IPTG (orange and blue lines, respectively).

Figure S3 - Original cytometry data for Figure 3.

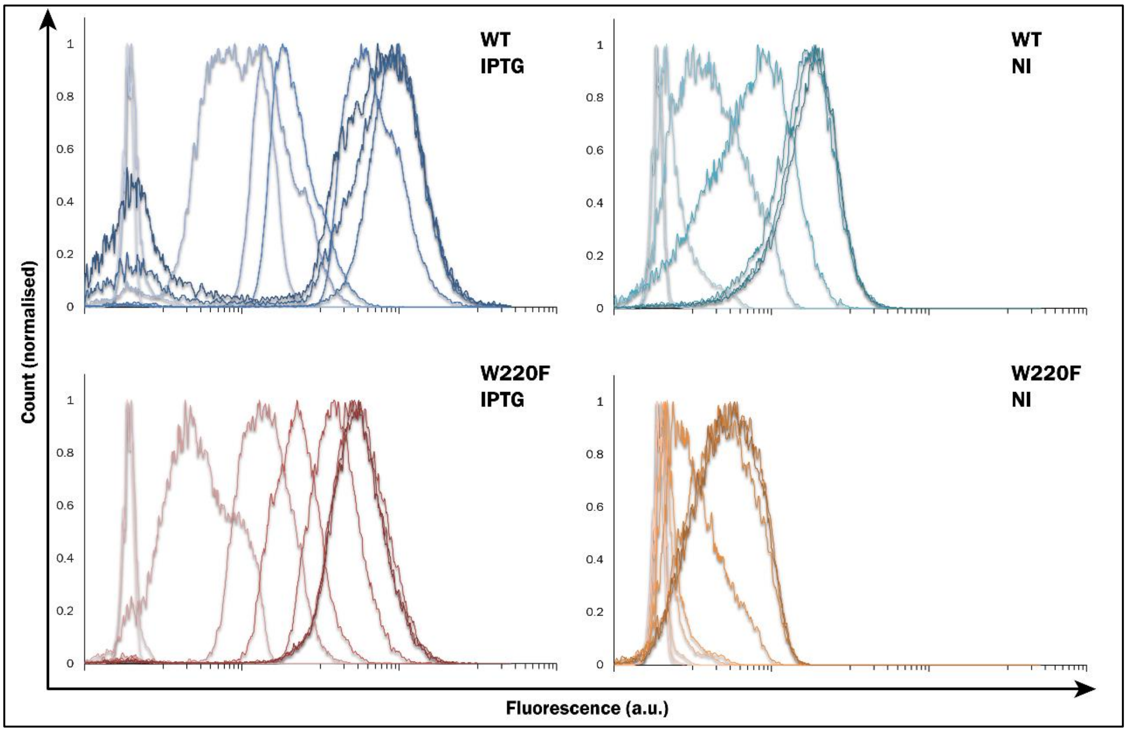

Raw cytometry data used to build the plots in Figure 3. The fluorescence distribution of cells bearing either pLIGFP (blue/cyan) or pLIGFP_W220F (red/brown) is shown for cultures incubated in the presence (left plots) or absence (right plots) of 0.01 mM IPTG. Cultures were sampled at the following time points: induction time (time 0), 1 h, 2 h, 5 h, 7 h, 10 h, 21 h, 24 h and 26 h (indicated by a colour gradient from light to dark). The decrease in GFP fluorescence observed at 24 h and 26 h in the case of the WT repressor (Figure 3) is due to the increase of a population of cells that do not produce GFP.

References

1. **DistancesRH** [http://pymolwiki.org/index.php/DistancesRH]
2. Daber R, Sochor M, Lewis M: **Thermodynamic analysis of mutant lac repressors.** *Journal of Molecular Biology* 2011, **409:**76-87.
